# The High-Fat Diet Based on Extra-Virgin Olive Oil Causes Dysbiosis Linked to Colorectal Cancer Prevention

**DOI:** 10.3390/nu12061705

**Published:** 2020-06-07

**Authors:** Carmen Rodríguez-García, Cristina Sánchez-Quesada, Ignacio Algarra, José J. Gaforio

**Affiliations:** 1 Department of Health Sciences, Faculty of Experimental Sciences, University of Jaén, 23071 Jaén, Spain; crgarcia@ujaen.es (C.R.-G.); csquesad@ujaen.es (C.S.-Q.); ialgarra@ujaen.es (I.A.); 2 Center for Advanced Studies in Olive Grove and Olive Oils, University of Jaen, Campus las Lagunillas s/n, 23071 Jaén, Spain; 3 Agri-Food Campus of International Excellence (ceiA3), 14071 Córdoba, Spain; 4 CIBER Epidemiología y Salud Pública (CIBER-ESP), Instituto de Salud Carlos III, 28029 Madrid, Spain

**Keywords:** inflammation, sunflower oil, coconut oil, gut microbiome, *Akkermansia*, *Neisseria*, *Staphylococcus*, firmicutes, bacteroidetes, *Prevotella*

## Abstract

The present study aims to examine the effects of three different high-fat diet (HFD) on mice gut microbiota in order to analyse whether they create the microenvironmental conditions that either promote or prevent colorectal cancer (CRC). We evaluated colonic mucosa-associated microbiota in CD1 mice fed with HFD, based on 60% kcal from fat-containing coconut, sunflower or extra-virgin olive oil as the only source of fat. The main findings were as follows: (a) All HFD produced a decrease in the richness and diversity of the intestinal microbiota that was independent of mouse weight, (b) HFD switched *Lactobacillus* to *Lactococcus*. In general, the results showed that both sunflower- and coconut-HFD generated a pro-inflammatory intestinal microenvironment. In brief, coconut-HFD decreased *Akkermansia* and increased *Staphylococcus, Prevotella and Bacteroides* spp. abundance. Sunflower-HFD reduced *Akkermansia* and *Bifidobacterium*, while enhancing *Sphingomonas* and *Neisseria* spp. abundance. In contrast, EVOO-HFD produced an anti-inflammatory microenvironment characterised by a decreased *Enterococcus*, *Staphylococcus*, *Neisseria* and *Pseudomonas* spp. abundance. At the same time, it increased the Firmicutes/Bacteroidetes ratio and maintained the *Akkermansia* population. To conclude, EVOO-HFD produced changes in the gut microbiota that are associated with the prevention of CRC, while coconut and sunflower-HFD caused changes associated with an increased risk of CRC.

## 1. Introduction

Nowadays, colorectal cancer (CRC) is among the most common and deadly neoplasms [1]. It is assumed that both, a Western-style diet with high-fat content and a sedentary lifestyle are responsible for the increase in their global incidence [1]. In fact, CRC is 3–4 times more common in developed than in developing nations. High fat intake is one of the characteristics of the Western pattern diet, and this correlates with an increased risk of CRC [2]. The trigger mechanism by which the risk increases is not yet well known, but it has been proposed that it is due to a change in the intestinal microbiota that favour a low-intensity inflammatory process [3].

Gut microbiota is a highly complex ecosystem, with great individual variations and thousands of microbial species. The most predominant phylum in the healthy gut are Firmicutes and Bacteroidetes, followed by Actinobacteria, Proteobacteria and Verrucomicrobia [4]. Under normal physiological conditions, gut microbiota regulate fat metabolism (by bile acids biotransformation), synthesise essential amino acids and vitamins, and facilitate the digestion of complex plant carbohydrates into short-chain fatty acids (mainly butyrate, propionate and acetate). The gut microbiome contributes significantly to host immunity and metabolic homeostasis [5,6]. However, alterations in the mucosa, together with an unfavourable genetic predisposition, favour the growth of opportunistic microbes that promote functional and morphological changes (dysbiosis) and lead to chronic inflammation. Chronic inflammation, in turn, contributes to dysbiosis [7]. Pro-inflammatory cytokines and opportunistic pathogens affect the epithelial integrity, and chronic inflammation together with oxidative stress leads to the loss of the epithelial barrier, and may result in a vicious cycle of immune hyperactivation and aggravation of barrier dysfunction [8]. In addition, defects in the host barrier enhance permeability and promote the invasion of harmful bacteria, which may lead to bacterial translocation across the epithelial layers [5], leading to the development of CRC [2].

Although high-fat diets (HFD) have been identified as promoting the development of CRC [2], not all types of fats have the same health effect. While animal fats have harmful effects on inflammation and CRC development, the effects of edible vegetable oils on gut microbiota dysbiosis have not been studied sufficiently yet [9]. All edible vegetable oils are assumed to be healthy, but given their different fatty acid profile and minor compounds composition, their impact on gut microbiota is expected to be different.

Since HFD is typically present in Western-pattern diets and is a risk factor of CRC, the present study aims to examine, in an experimental murine model, the influence of three HFD, each one prepared with different edible vegetable oils (coconut oil, sunflower oil and extra virgin olive oil) on gut microbiota.

## 2. Materials and Methods

### 2.1. Experimental Animals

Female CD1 mice were purchased from Charles River Laboratories (Wilmington, MA, USA). Four-week-old mice (*n* = 44) were housed (5–6 per cage) in ventilated racks and cages with environmental control (temperature: 20  ±  2 °C; humidity: 55–65%; 12 h light/12 h dark cycle).

Animal care and experiments were conducted following the guidelines of the Spanish Society for Laboratory Animal Science. The procedures applied to these animals were approved by the Ethical Committee of the University of Jaen (Record number: CEEA-100217-1) and the Ethical Committee of Animal Experiments of Regional Ministry of Agriculture, Fishing and Environment of Regional Government of Andalusia, Spain (Approval number: 16/03/2017/044).

### 2.2. Diets

The mice were fed with maintenance chow diet (defined as chow diet), with 13% kcal from fat (Ref. 2014S), an intermediate fat diet with 22% kcal from fat (Ref. 2019S) and custom basis (Ref. TD.170709) ready to use (fat free) for HFD with 60% kcal from fat were purchased from ENVIGO^®^ (Madison, WI, USA). The custom basis fat free was exclusively made by ENVIGO^®^ for the present study. In order to prepare each one of the HFD, we added one of the following oils to the custom basis: extra virgin olive oil (EVOO), coconut oil and sunflower oil. The HFD were made and administered daily in sterile conditions (Tables S1 and S2).

### 2.3. Experimental Design

On arrival, the mice were randomly divided into four groups (*n* = 11 each), and they were assigned to a different diet:

Group 1: Chow diet (chow)
Group 2: HFD of coconut oil (coconut-HFD)
Group 3: HFD of sunflower oil (sunflower-HFD)
Group 4: HFD of extra virgin olive oil (EVOO-HFD)

**Figure 1:**
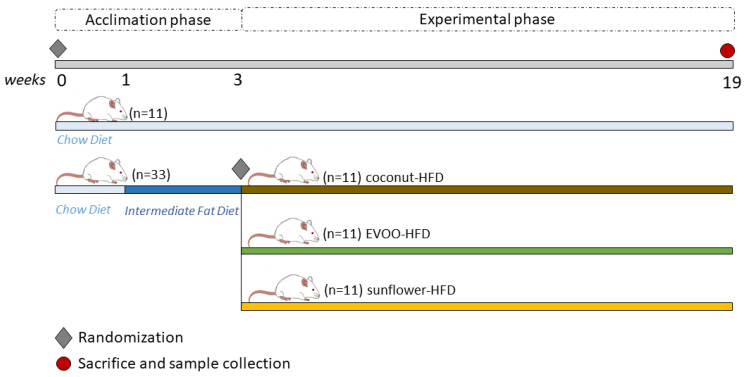
Experimental design.

Before the dietary intervention, the mice were kept in an acclimation phase for three weeks. In the first week, all the mice were fed with chow diet, and the following two weeks, the chow group continued on the same diet, but HFD groups were provided with an intermediate fat diet. Once the intervention phase started, the mice were fed either a chow diet, EVOO, coconut or sunflower HFD with ad libitum access to water and food for sixteen weeks (Figure 1).

Body weight and indirect food intake were measured weekly. The mice were then sacrificed using a euthanasic mixture of ketamine (160 mg/kg) and xylazine (10 mg/kg). The intestine was completely dissected, freshly cut and stored at −80 °C until analysis.

### 2.4. Sample Collection and Mucosa-Associated Microbiota Analysis by PCR Amplification and Sequencing of the 16S rRNA Gene

Thirty-two distal colon samples were analysed, corresponding to eight mice randomly selected from each experimental group. Samples were carefully dissected, and the colonic mucosa-associated microbiota was extracted and lysed. The nucleases were inactivated through mechanic disruption and enzymatic treatment. Genomic DNA was isolated from the bacterial colonies and purified with the QIAamp PowerFecal DNA kit (Qiagen, Hilden, Germany) following the manufacture’s protocol. 

The quality and quantity of the bacterial colonies were analysed by spectrophotometry using Nanodrop (Thermo Scientific, Waltham, MA, USA). DNA amplification of the 16S rRNA V3-V4 region of the bacteria’s rRNA genes was carried out with a two-step PCR protocol, using the PCR primers recommended by Klindworth et al. [10]. The obtained products were verified with the PicoGreen-based DNA quantification assay (Table S3). Sequencing of the gut microbiota was performed with Illumina Miseq (Novogene, Beijing, China). Sequencing was performed using the manufacturers’ protocol with a 300 pb pair-end design and 50,000 to 100,000 readings per sample were obtained. A positive control was included and another negative control was included during sequencing [11]. In both cases the result was optimal, the positive control had the expected species and the negative control had less than 50 sequences.

### 2.5. Bioinformatics Analysis

PEAR V.0.9.1 software (The Exelisis Lab, Heidelberg, Germany) was used to join each pair of sequences (R1 and R2) coming from the sequencing platform, taking into account a minimum overlap of 70 nts at each end, in this way a unique and complete sequence was obtained. Then, using the Cutadapt v1.8.1 program, the sequencing adapters of both ends present in each sample were eliminated.

Once the sequences without adapters were obtained, those readings that were below Q20 and less than 100 pb in length were eliminated. The reformat module of BBMap v38 was used to perform this analysis; this programme also cut out those nucleotide bases at both ends that presented a value of quality lower than that indicated (Q20). The last step in quality processing was the elimination of possible chimaera sequences resulting from incomplete extension during amplification by the PCR method. This step was carried out with the Uchime programme, which allows the detection and elimination of these amplicons from a reference database (ChimeraSlayer).

The CDHit v4.8.1 program (Weizhong Li’s Group, La Jolla, CA, USA) was used to determine the microbial diversity present in the sample, grouping sequences with a similarity threshold of 97% or higher in operational taxonomic units (OTUs). Each OTU was compared against the RefSeq 16S rRNA gene database (NCBI) using the BLAST tool. 

For each of the samples, a rarefaction curve was performed, reaching a saturation situation of detection (plate), to corroborate that all the organisms had been detected (Figure S1). Shannon–Wiener and Chao1 biostatistical values were calculated to estimate the specific biodiversity present in the sample and the total number of species. The local contribution to beta diversity analysis (LCBD) was done representing the degree of uniqueness of the sampling units in terms of community composition and based on the number of the standardized number of counts for rarefaction.

### 2.6. Statistical Analysis

The analysis consisted of a differential study of the population using the DESeq2 R tool focused on microbiomes. To compare data, bacteria with minor than 0.01% of presence were eliminated. In addition, principal coordinate analysis, canonical correspondence, beta dispersion analysis of the samples, and a differential study of diversity were performed. To show the effect of different diets on the relative abundance of taxa, a PERMANOVA test was performed. *p*-values adjusted by FDR has been used in DESesq2 test. Data are represented as mean ± SD and *p* values less or equal to 0.05 were considered statistically significant.

## 3. Results

### 3.1. High-Fat Diets Promoted Dysbiosis Independently of Body Weight and the Type of Vegetable Fat Present in the Diet

**Figure 2:**
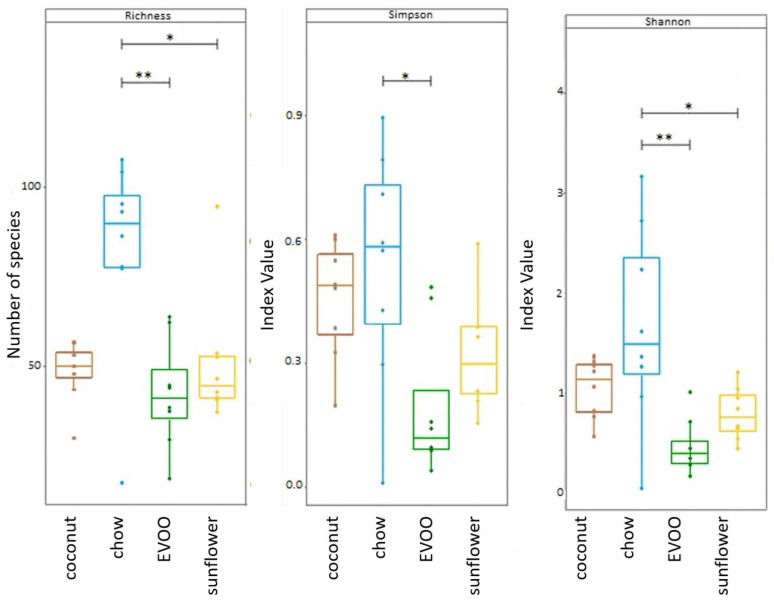
Gut microbiota richness and diversity (Shannon and Simpson) (evaluated by Chao-1). Boxplot figure representing the diversity of the samples according to the variables studied * *p* < 0.05 and ** *p* < 0.01 (ANOVA test).

Regardless of the type of vegetable fat used, HFD led to a decrease in the richness and diversity (Simpson and Shannon test, evaluated by Chao-1) of gut microbiota. Diversity reduction for EVOO- and sunflower-HFD were statistically significant (*p* < 0.05 for both EVOO- and sunflower-HFD) (Figure 2).

**Figure 3:**
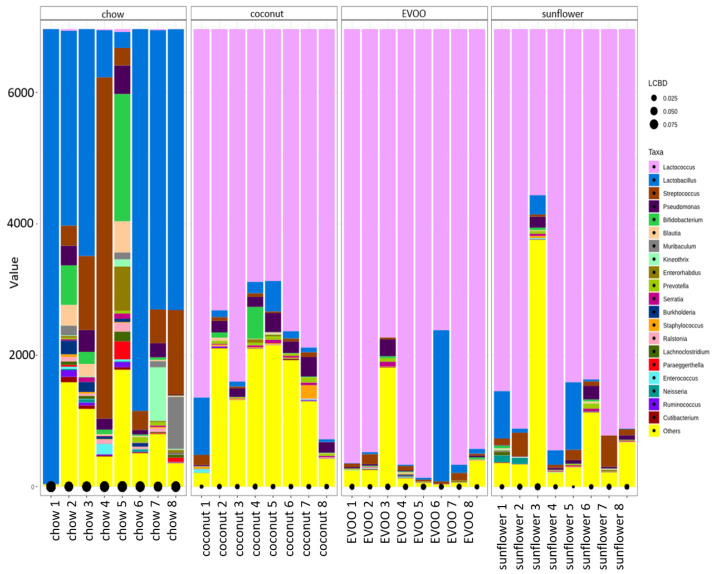
The local contribution to beta diversity analysis. LCBD values represent the degree of uniqueness of the sampling units in terms of community composition. Accumulative bar graph comparing the genera detected in the samples with the 16S rRNA-based bacteria profiles.

The local contribution to the beta diversity test verified that each group’s sample contribution to diversity was relatively homogeneous within the group (Figure 3). 

**Figure 4:**
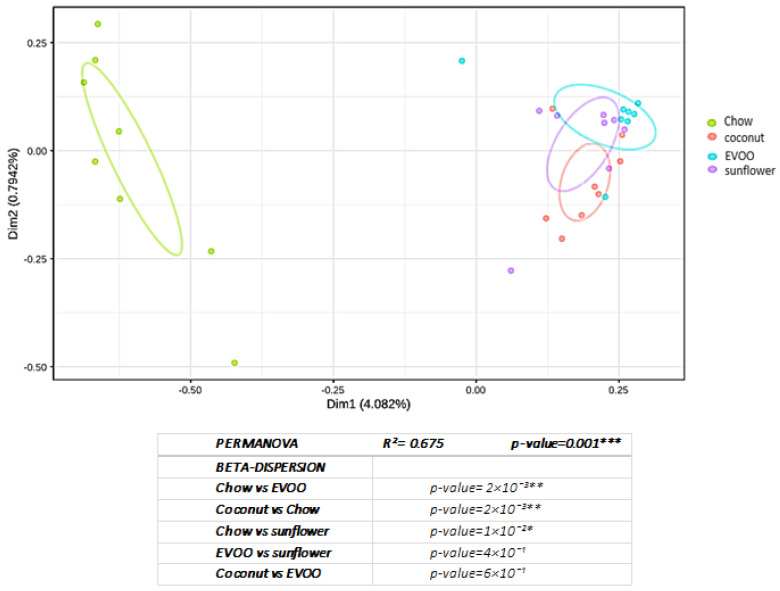
The principal coordinate analysis (PCoA) plot made with unweighted unifrac distances between samples of microbiome, grouped by diets. The main coordinate graph showing the differences between samples and its group, with 30% CI ellipses. A PERMANOVA analysis has also been carried out to detect whether the separation between groups is significant. * *p* < 0.05, ** *p* < 0.01, *** *p* < 0.001.

Principal coordinate analysis (PCoA) and PERMANOVA analysis corroborated that HFD promoted a change in the bacterial community in a significant way (*p* = 0.002 for coconut and EVOO-HFD, and *p* = 0.01 for sunflower-HFD) (Figure 4).

To compare if these changes in bacterial communities may be related to body weight, the initial and final mean weights of each group were analysed. Data showed that coconut-HFD increased body weight, likewise for the chow diet, while EVOO- and sunflower-HFD increased it notably (Table 1).

### 3.2. Main Findings Found at the Phylum Level: EVOO-HFD Specifically Reduced Proteobacteria While Coconut-HFD Decreased Verrucomicrobia

**Figure 5:**
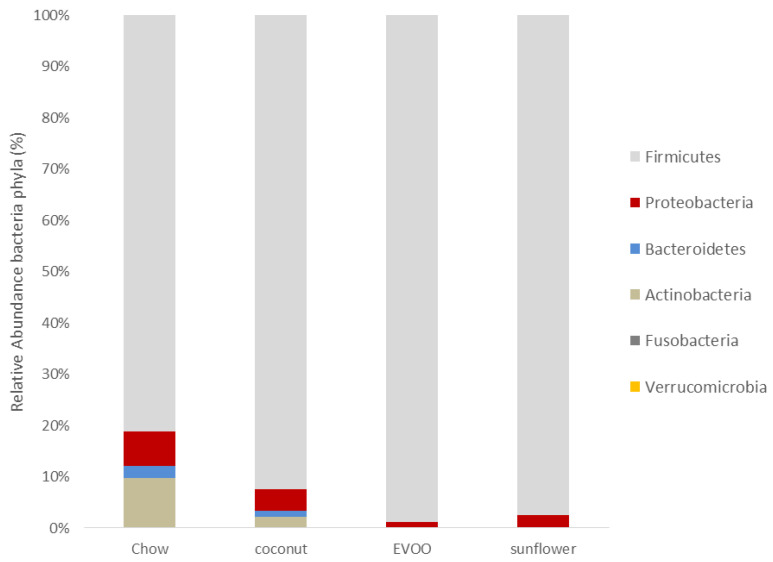
The relative abundance of bacterial phyla (%). The taxonomic summary bar plots identification of the microbiota at the phyla level.

**Figure 6:**
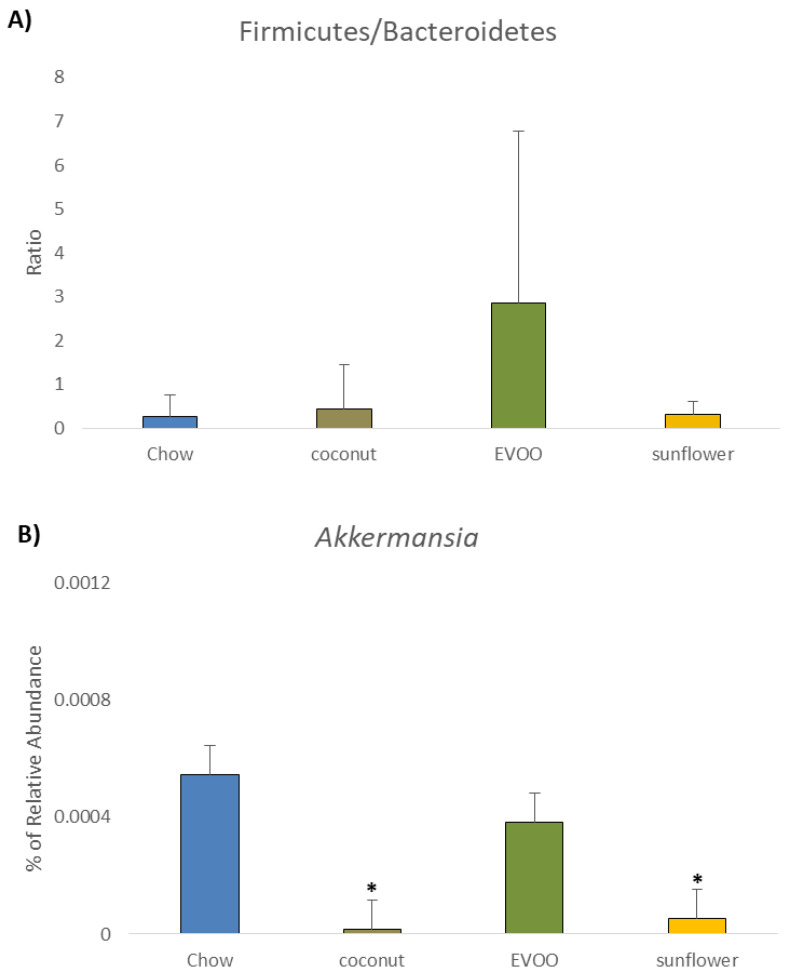
The relative abundance of bacterial phyla and genera (%). (**A**) Firmicutes/Bacteroidetes Ratio. (**B**) *Akkermansia*. * *p* < 0.05 for HFD (coconut and sunflower) vs. chow diet.

At the phylum level, the abundance of mice gut microbiota was different between chow and all three HFD groups (Figure 5 and Table 2). However, Firmicutes remained in all diets as the majority phylum. Although there were no statistical differences between groups, EVOO- and sunflower-HFD showed an increase in this phylum. With regard to Bacteroidetes, all HFD reduced this phylum in a significant way in comparison with the chow group (*p* < 0.05). Figure 6A shows that the Firmicutes/Bacteroidetes ratio increased in EVOO-HFD while it remained almost unchanged in both coconut- and sunflower-HFD. As in Bacteroidetes, all the HFD decreased the Actinobacteria phylum in a significant way. Despite the fact that no statistical differences between HFD groups were observed, EVOO- and coconut-HFD acted differently; microbiota from EVOO-HFD mice suffered a reduction in Proteobacteria (*p* <0.001), while coconut-HFD decreased Verrucomicrobia (*p* < 0.01). Figure 5 illustrates the taxonomic summary bar plots identification of the microbiota at the phylum level, concluding that all HFD promoted dysbiosis on gut microbiota.

### 3.3. Main Findings Found at Both Genera and Species Levels: HFD Produced Both Switching Lactobacillus for Lactococcus and Only Coconut-HFD Promoting Staphylococcus Colonisation

**Figure 7:**
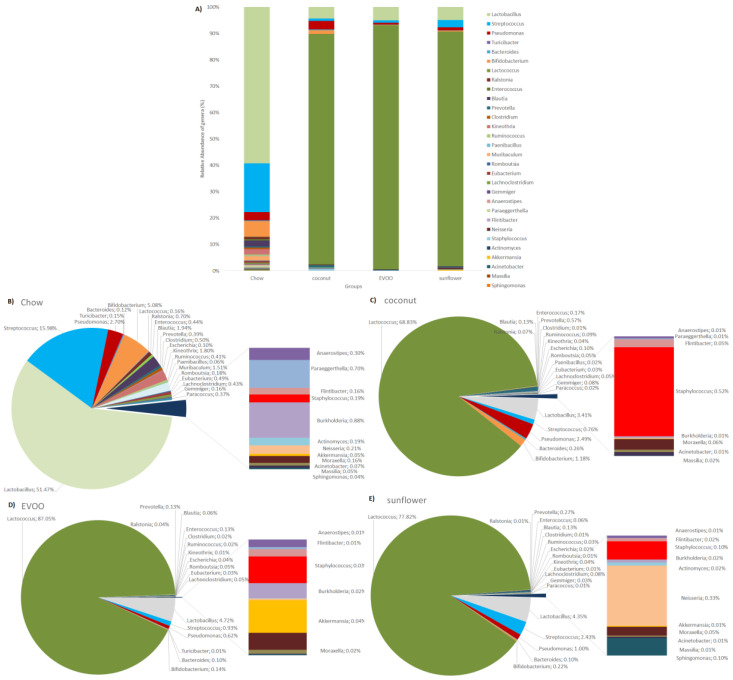
The relative abundance of bacteria genera (%). (**A**) The taxonomic summary bar plots at the genera level, (**B**) chow, (**C**) coconut, (**D**) EVOO, (**E**) sunflower.

Despite decreasing diversity in all HFD groups in comparison with chow, there were some relevant changes regarding some bacterial genera and species (Table 3 and Table 4). The most relevant change induced by HFD was *Lactobacillus* reduction (mainly *Lactobacillus reuteri*) and *Lactococcus* increase (mostly *Lactococcus lactis*) (Figure 7). HFD also decreased *Streptococcus*, *Turicibacter*, *Blautia*, *Clostridium*, *Ruminococcus* and *Anaerostipes* genera in a significant way in comparison with chow (*p* < 0.05). Differences between chow vs. EVOO- and sunflower-HFD were observed, reducing *Enterococcus* in a significant way (mainly *Enterococcus gallinarum*). Regarding differences between HFD groups, EVOO-HFD (0.031 ± 0.04% of relative abundance) and coconut-HFD (0.522 ± 0.82% of relative abundance) showed statistical differences with respect to *Staphylococcus* genera (*p* < 0.05). Coconut enhanced the relative abundance of *Staphylococcus epidermidis*.

### 3.4. Sunflower-HFD Enhanced Sphingomonas Genus

Regarding Proteobacteria, mice of the EVOO-HFD group presented a significant decrease in *Pseudomonas* abundance; in this genus, EVOO-HFD mainly reduced *Pseudomonas migulae* (Table 3 and Table 4). In comparison to EVOO- and coconut-HFD, sunflower-HFD enhanced *Neisseria*, especially *Neisseria mucosa* sp. (*p* < 0.05), while sunflower-HFD significantly increased *Sphingomonas* in comparison to chow, coconut-HFD and EVOO-HFD (Figure 7).

### 3.5. Coconut and Sunflower-HFD Reduced Akkermansia Muciniphila Abundance

With regard to the Bacteroidetes phylum, all the HFD decreased Muribaculum abundance (Table 3). For Actinobacteria, all the HFD decreased *Bifidobacterium*, but only EVOO- and sunflower-HFD in a significant way.

Figure 6B shows that for the Verrucomicrobia phylum, coconut and sunflower-HFD reduced *Akkermansia* (mainly *Akkermansia muciniphila*) abundance in comparison with chow (*p* < 0.05) (Table 4).

## 4. Discussion

Western diets characterised by high fat intake have been strongly linked to CRC in several epidemiological studies [12,13]. Indeed, diet can modulate the gut microbiota characteristics, which can eventually lead to an increased or decreased risk of CRC [14].

For the first time, our results showed that the presence of EVOO as the only source of fat in HFD, caused a dysbiosis that could be associated with the prevention of CRC, while coconut and sunflower-HFD produced a dysbiosis associated with an increased CRC risk. Although all mice were fed ad libitum during the 16 weeks of dietary intervention, surprisingly mice fed with the coconut-HFD showed a similar weight to mice of the control group (Table 1). By contrast, mice fed with the EVOO or sunflower-HFD gained considerable weight at the end of the dietary intervention. The results clearly show that HFD of coconut oil, EVOO or sunflower oil produce intestinal dysbiosis. Although similar results have been described by other researchers [15], the present study is the only one that has used HFD containing a single type of oil as the sole source of fat; this is important because not all fats have the same impact on health. Indeed, as we can see in Figure 2, both the richness and diversity of the gut microbiota are diminished by each of the three diets administered. Similarly, beta diversity analyses corroborate these results (Figure 3). In line with these results, both the principal coordinated analysis (PCoA) and PERMANOVA analysis show a significant change in the gut microbiota of mice fed any of the three diets studied (Figure 4).

Firmicutes were the most abundant phylum found in mice gut microbiota fed with chow diet; this is consistent with other research findings [16]. Both the sunflower and EVOO-HFD diets increased the percentage of Firmicutes, although not in a statistically significant way (Table 2 and Figure 5). Interestingly, we found that EVOO-HFD increased the Firmicutes/Bacteroidetes ratio (Figure 6A). Although the increase in this ratio has been correlated with obesity, it is also associated with CRC prevention through the modulation of the inflammatory process [17,18]. 

Gut microbiota of all HFD studied, decreased *Lactobacillus reuteri* and promoted an increase in *Lactococcus lactis* (Table 4 and Figure 7A). Both species are probiotics that have a key role in modulating inflammatory processes. *L. reuteri* is able to improve immune activity to combat autoimmune diseases, inhibiting inflammation by reducing Th1/Th2 cell ratio and their associated cytokines [19]. On the other hand, *L. lactis* plays an important role in the antioxidant defence of gut microbiota and in the maintenance of the pro- and anti-inflammatory balance [20,21]. 

*Bifidobacterium* are a group of probiotic bacteria involved in normal colonocyte maintenance [22]. Our results showed that all three HFD produced a decrease in *Bifidobacterium*, although this only has statistical significance for EVOO- and sunflower-HFD (Table 3 and Figure 7A). 

*Akkermansia muciniphila* has been associated with numerous human health benefits, for example, with lifespan [23]. A decrease in this bacterium has been found in individuals with inflammatory bowel disease, ulcerative colitis and Crohn’s disease. Its administration can also decrease inflammatory cytokines levels in intestinal inflammation [24]. Other authors have reported that the food additive carrageenan decreases *A. muciniphila* and promotes both inflammation and colitis in experimental animals [25].

On the contrary, some phenolic compounds such as resveratrol and caffeic acid were able to restore *A. muciniphila* after colitis induction in mice, ameliorating gut inflammation [26,27]. Interestingly, our experimental study showed that coconut- and sunflower-HFD decreased the relative abundance of *A. muciniphila* significantly, while the EVOO-HFD maintained the population of this bacterium (Table 4 and Figure 6B). A possible hypothesis is that minority compounds found in EVOO may be involved in facilitating *A. muciniphila’s* survival. It is important to note that these minority compounds are not present in either coconut or sunflower oil.

Furthermore, we have shown that the presence of opportunistic gut pathogens linked to an increase of CRC risk are also altered according to the type of diet consumed. At the genus level, we identified that Streptococcus and *Clostridium* decreased their abundance in mice fed any of the high-fat diets. Some species of *Clostridium*, such as *C. difficile*, produce toxic bacterial enzymes involved in bacteraemia and CRC development [28,29]. Interestingly, the coconut-HFD increased *Staphylococcus* spp., although not in a statistically significant way (Table 3 and Figure 7C). On the contrary, the EVOO-HFD significantly decreased the abundance of *Staphylococcus* spp. and especially *Staphylococcus epidermidis* (Table 3 and Figure 7D). It is well known that *S. epidermidis* is capable of forming biofilms on the colon, enhancing CRC risk [30]. All the bacteria mentioned above are considered to be opportunistic pathogens and have been found on colon specimens from patients with colitis and autoimmune diseases [31].

Another interesting finding was that EVOO- and sunflower-HFD reduced *Enterococcus gallinarum* abundance in a significant way. *E. gallinarum* is considered a pathobiont able to translocate to the liver and other tissues, triggering autoimmune responses [32]. In addition, it has been suggested that antibiotic treatment prevents mortality in mice by suppressing the growth of *E. gallinarum* in tissues [33], suggesting that EVOO- and sunflower-HFD could act on the autoimmune response by controlling the abundance of *E. gallinarum* in the colon.

Several studies have demonstrated an increased abundance of Proteobacteria phylum in pathologies, including inflammatory bowel disease; in fact, inflammation represents a core aspect of pathologies associated with Proteobacteria [34]. In addition, its high abundance has been correlated with oxygen reactive species generation and inflammation in humans with inflammatory bowel disease, colitis and CRC [35,36,37]. Sunflower-HFD enhanced *Neisseria* and *Sphingomonas* significantly (Table 3 and Figure 7E), both of which are considered pathobionts [38]. In fact, its increase has been correlated with a pro-inflammatory response in the mucosa of the colon, promoting natural killer T-cell activity of colitis-associated cancers [39,40]. On the contrary, coconut-HFD reduced *Neisseria* abundance, and EVOO-HFD reduced not only *Neisseria* and *Pseudomonas*, but also Proteobacteria abundance (Table 3, Figure 7C,D). To our knowledge, there are no studies that have described the beneficial or harmful effects related to Proteobacteria induced by EVOO, coconut or sunflower-HFD, however, some authors have described the protective effects of EVOO on immunomodulation in murine experimental ulcerative colitis and patients with ulcerative colitis [41,42], that could be associated not only with inflammatory processes involved on the MAPK and NFκB signalling pathways but also with microbiota modulation, as our results showed [43]. 

In another pathogenic microbiome, *Prevotella* and *Bacteroides* abundance appeared to be higher in coconut-HFD than EVOO- and sunflower-HFD (Table 3 and Figure 7C). Both genera have negative effects on colitis through the enhancement of inflammation; in fact, its reduction ameliorates colonic inflammation [44,45]. *Prevotella* abundance is also related to the increase in epithelial inflammation and triggers autoimmune diseases [46,47]. The results suggest that coconut-HFD could produce an increased risk of CRC by stimulating the growth of *Prevotella* and *Bacteroides*, while diets rich in EVO or sunflower oil could have a protective effect. Different research with honey polyphenols, essential oi and different vegetable oils, have shown that they were able to improve oxidative stress resistance and intestinal inflammation by Bacteroidetes reduction [48,49]. We could hypothesise that the effect of EVOO is probably due to the presence of polyphenols that characterise this fat of vegetable origin.

**Figure 8:**
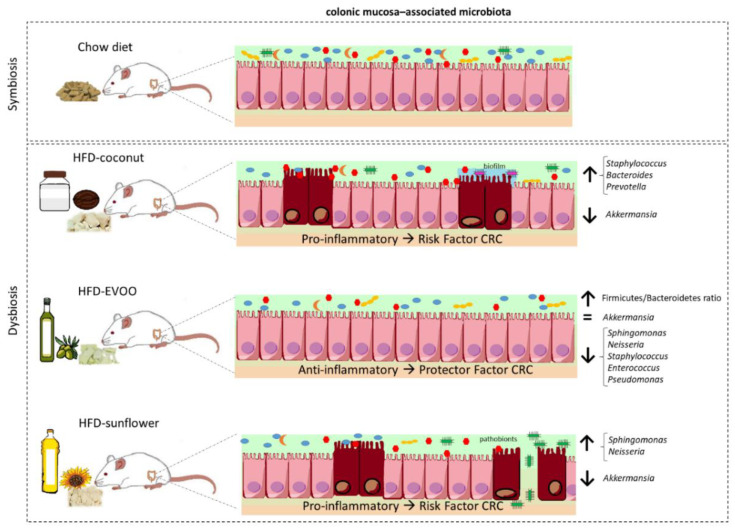
Summary of the main effects on gut microbiota caused by chow diet and HFD bases on coconut, EVOO or sunflower oil.

**Figure 9:**
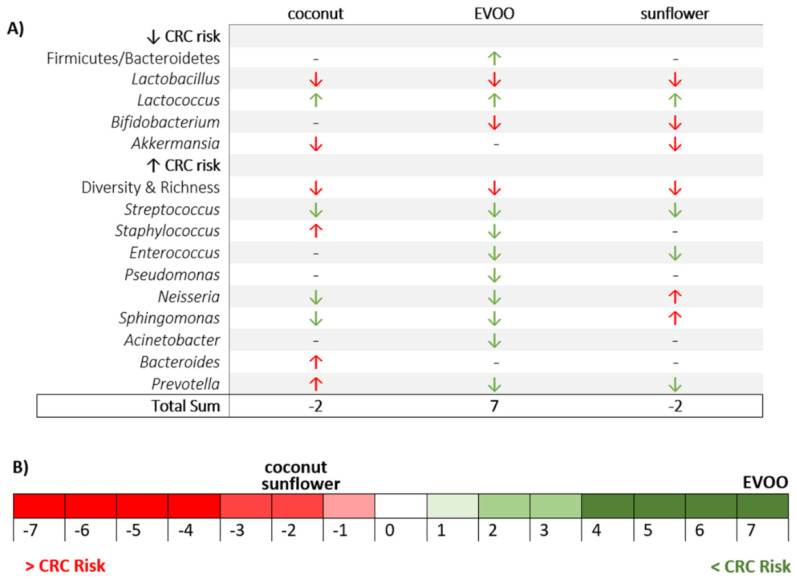
The effect of high-fat diets of sunflower, coconut and EVOO oils on dysbiosis linked to CRC risk. (**A**) Summary of HFD effects on bacteria related with CRC risk (Red arrow, increases CRC risk (minus 1); Green arrow, decreases CRC risk (plus 1); Dash, no changes (equal)). (**B**) Risk scale of developing CRC based on vegetable oil-HFD intake. Data are based on the total sum of Figure 9A.

The gut microbiota can modulate the inflammatory processes that can eventually result in the presence of a pro- or anti-tumour environment in the colon. Thus, the main objective of this study was to determine whether an HFD containing only EVOO, sunflower oil or coconut oil modifies the gut microbiota in a way that favours or prevents CRC. In brief, the main findings of this study could be summarised as follows: (a) the three HFD studied (EVOO-, sunflower- and coconut-HFD) produce a decrease in both the richness and diversity of the gut microbiota, (b) these alterations do not correlate with body weight at the end of the dietary intervention, (c) each of these HFDs has a different impact on the gut microbiota by promoting or inhibiting the growth of different bacteria that are associated with a pro- or anti-inflammatory environment (summarised in Figure 8), and that are associated with the dysbiosis linked to colorectal cancer risk (Figure 9) 

This study has several limitations, including the sample size, the exploratory nature of the study, and type-II errors which could fail to detect effects present in small-scale studies. For instance, further studies are needed to assert these hypotheses. The strength of this study is that we use a single source of fat in the diet; a large number of studies use high-fat diets supplemented with different types of fat. This can be misleading, since the basis of nutritional preparations for animals has a significant amount of both vegetable (soy oil) and animal (lard) fat. Giving therefore results that could be wrong or a mixed of effect from animal and vegetable oil. Using a single type of fat allows us to observe its specific effect on health.

Finally, it is difficult to extrapolate the results obtained in this study to humans due to interspecies variability. However, the usefulness of studies using murine experimental models as a preclinical model is widely accepted [50]. In addition, it is not possible to conduct a dietary intervention study in humans in which the diet contains only one type of fat, highlighting the importance of the data obtained in this study.

## Figures and Tables

**Table 1:** Initial and final body weights of mice fed with chow and HFD.

Weight	Chow	Coconut	EVOO	Sunflower
Initial (g)	29.49 ± 0.37	28.65 ± 0.47	29.42 ± 0.76	31.04 ± 0.77
Final (g)	45.48 ± 1.85	44.88 ± 1.47	55.89 ± 2.65 **	55.3 ± 3.33 **

Data are shown as Mean ± SD of bodyweight (*n* = 11). ANOVA test, ** *p* < 0.01 for HFD (EVOO, sunflower) vs. chow diet.

**Table 2:** Percentage of relative abundance of Phylum.

Phylum	Chow	Coconut	EVOO	Sunflower
Firmicutes	78.695 ± 20.27	75.014 ± 13.08	93.739 ± 10.56	86.081 ± 18.37
Proteobacteria	6.578 ± 5.34	3.447 ± 1.86	0.958 ± 1.61 ***	2.17 ± 1.35
Bacteroidetes	2.331 ± 3.04	0.948 ± 0.49 *	0.283 ± 0.48 ***	0.532 ± 0.46 ***
Actinobacteria	9.186 ± 15.32	1.712 ± 2.78 **	0.437 ± 0.22 ***	0.654 ± 0.45 ***
Fusobacteria	0.083 ± 0.14	0.011 ± 0.02	0.007 ± 0.01	0.046 ± 0.09
Verrucomicrobia	0.055 ± 0.12	0.002 ± 0.003 **	0.038 ± 0.06	0.011 ± 0.02

Data are shown as Mean ± SD and were analysed by the PERMANOVA test. * *p* < 0.05, ** *p* < 0.01, *** *p* < 0.001 for HFD (coconut, EVOO, and sunflower) vs. chow diet. (*n* = 8).

**Table 3:** Percentage of relative abundance of Genera.

Genera	Chow	Coconut	EVOO	Sunflower
FIRMICUTES				
*Lactobacillus*	51.471 ± 32.92	3.412 ± 3.99 ^1^	4.717 ± 11.76 ^1^	4.351 ± 5.58 ^1^
*Streptococcus*	15.982 ± 24.53	0.76 ± 0.57 ^1^	0.928 ± 0.66 ^1^	2.428 ± 2.35 ^1^
*Turicibacter*	0.152 ± 0.23	0.001 ± 0.003 ^1^	0.006 ± 0.006 ^1^	0.004 ± 0.004 ^1^
*Lactococcus*	0.156 ± 0.19	68.83 ± 12.28 ^1^	87.055 ± 13.74 ^1^	77.819 ± 17.58 ^1^
*Enterococcus*	0.444 ± 0.84	0.173 ± 0.4	0.125 ± 0.24 ^1^	0.06 ± 0.06 ^1^
*Blautia*	1.942 ± 2.71	0.129 ± 0.18 ^1^	0.058 ± 0.06 ^1^	0.129 ± 0.12 ^1^
*Clostridium*	0.505 ± 0.64	0.01 ± 0.02 ^1^	0.024 ± 0.02 ^1^	0.01 ± 0.02 ^1^
*Kineothrix*	1.796 ± 3.8	0.036 ± 0.07 ^1^	0.014 ± 0.02 ^1^	0.037 ± 0.05 ^1^
*Ruminococcus*	0.411 ± 0.48	0.094 ± 0.11 ^1^	0.023 ± 0.03 ^1^	0.034 ± 0.03 ^1^
*Paenibacillus*	0.061 ± 0.11	0.022 ± 0.05	0.003 ± 0.004 ^1^	0.0003 ± 0.0007 ^1^
*Romboutsia*	0.178 ± 0.24	0.046 ± 0.06 ^1^	0.048 ± 0.06	0.014 ± 0.02 ^1^
*Eubacterium*	0.495 ± 0.59	0.034 ± 0.05 ^1^	0.03 ± 0.06 ^1^	0.013 ± 0.01 ^1^
*Lachnoclostridium*	0.426 ± 0.47	0.053 ± 0.07 ^1^	0.046 ± 0.05 ^1^	0.079 ± 0.2 ^1^
*Gemmiger*	0.157 ± 0.23	0.081 ± 0.19	0.004 ± 0.004 ^1^	0.033 ± 0.05
*Anaerostipes*	0.296 ± 0.45	0.012 ± 0.02 ^1^	0.009 ± 0.01 ^1^	0.014 ± 0.02 ^1^
*Flintibacter*	0.162 ± 0.23	0.047 ± 0.06	0.009 ± 0.01 ^1^	0.017 ± 0.02 ^1^
*Staphylococcus*	0.194 ± 0.16	0.522 ± 0.82	0.031 ± 0.04 ^1^^,^^2^	0.101 ± 0.1
PROTEOBACTERIA				
*Pseudomonas*	2.702 ± 2.23	2.489 ± 1.33	0.618 ± 1.19 ^1^	0.999 ± 1.09
*Ralstonia*	0.696 ± 0.57	0.071 ± 0.74 ^1^	0.038 ± 0.06 ^1^	0.014 ± 0.01 ^1^
*Escherichia*	0.095 ± 0.12	0.102 ± 0.04	0.038 ± 0.06	0.023 ± 0.02
*Paracoccus*	0.375 ± 0.98	0.024 ± 0.02	0.003 ± 0.004	0.006 ± 0.01
*Burkholderia*	0.882 ± 1.07	0.01 ± 0.02 ^1^	0.018 ± 0.05 ^1^	0.018 ± 0.05 ^1^
*Moraxella*	0.159 ± 0.35	0.063 ± 0.14	0.02 ± 0.04	0.049 ± 0.11
*Neisseria*	0.214 ± 0.24	0.004 ± 0.008 ^1^	0.002 ± 0.003 ^1^	0.335 ± 0.61 ^2^^,^^3^
*Acinetobacter*	0.073 ± 0.14	0.013 ± 0.01	0.005 ± 0.009 ^1^	0.007 ± 0.01
*Massilia*	0.049 ± 0.08	0.022 ± 0.06	0.0009 ± 0.002 ^1^	0.007 ± 0.01
*Sphingomonas*	0.036 ± 0.06	0.002 ± 0.005	0.001 ± 0.001	0.097± 0.17 ^2^^,^^3^
BACTEROIDETES				
*Bacteroides*	0.125 ± 0.13	0.264 ± 0.21	0.103 ± 0.2	0.099 ± 0.12
*Prevotella*	0.389 ± 0.48	0.57 ± 0.29	0.134 ± 0.24	0.273 ± 0.33
*Parabacteroides*	0.004 ± 0.003	0.017 ± 0.02	0.008 ± 0.01	0.008 ± 0.01
*Muribaculum*	1.509 ± 3.26	0.003 ± 0.007 ^1^	0.0003 ± 0.0007 ^1^	ND ^1^
ACTINOBACTERIA				
*Bifidobacterium*	5.075 ± 9.59	1.175 ± 2.52	0.138 ± 0.13 ^1^	0.224 ± 0.24 ^1^
*Paraeggerthella*	0.701 ± 1.38	0.005 ± 0.01 ^1^	0.003 ± 0.005 ^1^	0.002 ± 0.003 ^1^
*Actinomyces*	0.192 ± 0.47	0.0001 ± 0.0003 ^1^	0.0006 ± 0.001 ^1^	0.016 ± 0.03 ^1^
VERRUCOMICROBIA				
*Akkermansia*	0.055 ± 0.12	0.001 ± 0.003 ^1^	0.038 ± 0.06	0.005 ± 0.008 ^1^

Data are shown as Mean ± SD and were analysed by PERMANOVA test; ^1^ *p* < 0.05 for groups vs. chow diet; ^2^ *p* < 0.05 for groups vs. coconut; ^3^ *p* < 0.05 for groups vs. EVOO; Not detectable is shown as ND = 0.

**Table 4:** Percentage of Relative Abundance of Species.

Species	Chow	Coconut	EVOO	Sunflower
*Lactobacillus reuteri*	42.45 ± 29.95	0.335 ± 0.57 ^1^	1.106 ± 2.84 ^1^	0.658 ± 0.77 ^1^
*Lactobacillus taiwanensis*	5.979 ± 7.31	0.002 ± 0.003 ^1^	0.006 ± 0.01	0.002 ± 0.004 ^1^
*Lactobacillus animalis*	1.71 ± 2.29	1.449 ± 2.1	0.415 ± 0.45	1.533 ± 2.76
*Lactobacillus caviae*	0.501 ± 0.34	0.004 ± 0.01 ^1^	0.019 ± 0.05 ^1^	0.009 ± 0.01 ^1^
*Lactobacillus gasseri*	0.129 ± 0.16	0.017 ± 0.02	2.85 ± 8.05	0.019 ± 0.05
*Lactobacillus secaliphilus*	0.078 ± 0.14	0.0002 ± 0.0007 ^1^	0.023 ± 0.06	0.001 ± 0.002 ^1^
*Lactobacillus faecis*	0.026 ± 0.04	0.011 ± 0.02	0.01 ± 0.02	0.019 ± 0.03
*Lactobacillus johnsonii*	0.004 ± 0.004	1.351 ± 2.06	0.195 ± 0.38	2.012 ± 4.70
*Lactobacillus satsumensis*	0.002 ± 0.003	0.008 ± 0.01	0.016 ± 0.03	0.062 ± 0.12
*Streptococcus danieliae*	15.229 ± 24.65	0.711 ± 0.56 ^1^	0.76 ± 0.6 ^1^	1.774 ± 2.05 ^1^
*Streptococcus oralis*	0.255 ± 0.30	0.022 ± 0.03 ^1^	0.021 ± 0.04 ^1^	0.474 ± 1.11 ^2^^,^^3^
*Streptococcus sanguinis*	0.192 ± 0.43	0.0001 ± 0.0003 ^1^	0.006 ± 0.01 ^1^	0.055 ± 0.14
*Lactococcus lactis*	0.153 ± 0.19	68.407 ± 12.20 ^1^	86.28 ± 13.62 ^1^	77.241 ± 17.41 ^1^
*Lactococcus taiwanensis*	0.003 ± 0.002	0.419 ± 0.11 ^1^	0.761 ± 0.25 ^1^	0.557 ± 0.21 ^1^
*Enterococcus gallinarum*	0.361 ± 0.85	0.022 ± 0.06	0.003 ± 0.004 ^1^	0.028 ± 0.05 ^1^
*Blautia wexlerae*	0.613 ± 0.88	0.039 ± 0.06 ^1^	0.012 ± 0.01 ^1^	0.047 ± 0.07 ^1^
*Blautia luti*	0.559 ± 0.69	0.042 ± 0.09 ^1^	0.014 ± 0.02 ^1^	0.015 ± 0.02 ^1^
*Blautia obeum*	0.416 ± 0.90	0.012 ± 0.02 ^1^	0.002 ± 0.003 ^1^	0.004 ± 0.003 ^1^
*Blautia faecis*	0.045 ± 0.06	0.009 ± 0.01	0.001 ± 0.001 ^1^	0.005 ± 0.01 ^1^
*Clostridium scindens*	0.088 ± 0.09	0.014 ± 0.02	0.027 ± 0.04	0.029 ± 0.07
*Clostridium spiroforme*	0.101 ± 0.17	0.012 ± 0.03 ^1^	0.004 ± 0.01 ^1^	0.0004 ± 0.0005 ^1^
*Clostridioides difficile*	ND	0.001 ± 0.003	0.016 ± 0.03	0.003 ± 0.006
*Ruminococcus gnavus*	0.306 ± 0.8	0.021 ± 0.04 ^1^	0.024 ± 0.04 ^1^	0.051 ± 0.09 ^1^
*Eubacterium hallii*	0.38 ± 0.47	0.012 ± 0.02 ^1^	0.005 ± 0.01 ^1^	0.008 ± 0.01 ^1^
*Anaerostipes hadrus*	0.291 ± 0.45	0.012 ± 0.02 ^1^	0.009 ± 0.01 ^1^	0.014 ± 0.02 ^1^
*Staphylococcus epidermidis*	0.065 ± 0.09	0.328 ± 0.87	0.003 ^1^^,^^2^	0.031 ± 0.04 ^2^
*Pseudomonas migulae*	1.538 ± 1.56	1.199 ± 0.93	0.428 ± 0.89 ^1^	0.493 ± 0.58
*Pseudomonas trivialis*	0.277 ± 0.36	0.238 ± 0.20	0.075 ± 0.16	0.169 ± 0.25
*Pseudomonas helmanticensis*	0.521 ± 1.17	0.888 ± 1.64 ^1^	0.04 ± 0.1 ^1^	0.277 ± 0.76 ^1^
*Pseudomonas aeruginosa*	0.04 ± 0.07	0.001 ± 0.001 ^1^	0.001 ± 0.001 ^1^	ND ^1^
*Neisseria mucosa*	0.055 ± 0.15	0.003 ± 0.007 ^1^	0.0003 ± 0.0007 ^1^	0.176 ± 0.32 ^2^^,^^3^
*Ralstonia insidiosa*	0.685 ± 0.57	0.071 ± 0.07 ^1^	0.038 ± 0.06 ^1^	0.013 ± 0.01 ^1^
*Prevotella oralis*	0.003 ± 0.01	0.004 ± 0.01	0.005 ± 0.01	0.0002 ± 0.0007
*Prevotella copri*	0.217 ± 0.32	0.543 ± 0.27	0.113 ± 0.22	0.244 ± 0.32
*Muribaculum intestinale*	1.509 ± 3.26	0.003 ± 0.01 ^1^	0.0003 ± 0.0007 ^1^	ND ^1^
*Bifidobacterium longum*	0.683 ± 0.88	0.131 ± 0.22 ^1^	0.033 ± 0.05 ^1^	0.057 ± 0.08 ^1^
*Bifidobacterium animalis*	4.022 ± 8.49	0.124 ± 0.14 ^1^	0.094 ± 0.13 ^1^	0.12 ± 0.12 ^1^
*Bifidobacterium thermophilum*	0.053 ± 0.11	0.006 ± 0.02 ^1^	0.001 ± 0.001 ^1^	0.0001 ± 0.0003 ^1^
*Bifidobacterium adolescentis*	0.189 ± 0.21	0.096 ± 0.23	0.005 ± 0.005 ^1^	0.022 ± 0.04 ^1^
*Bifidobacterium bifidum*	0.052 ± 0.09	0.014 ± 0.03 ^1^	0.002 ± 0.005 ^1^	0.004 ± 0.01 ^1^
*Akkermansia muciniphila*	0.055 ± 0.12	0.001 ± 0.02 ^1^	0.038 ± 0.06	0.005 ± 0.01 ^1^

Data are shown as Mean ± SD and were analysed by PERMANOVA test; ^1^ *p* < 0.05 for groups versus chow diet; ^2^ *p* < 0.05 for groups versus coconut.; ^3^ *p* < 0.05 for groups versus EVOO.; Not detectable is shown as ND = 0.

## Supplementary Materials

The following are available online at https://www.mdpi.com/2072-6643/12/6/1705/s1, Figure S1: Rarefaction curves of the amplified samples for the detection of bacterial genera, Table S1: Composition of experimental high fat diets, Table S2: Minority compounds found in EVOO, Table S3: PicoGreen-Based DNA Quantification and Quality control analysis.
