# Supplementary material for: The High-Fat Diet Based on Extra-Virgin Olive Oil Causes Dysbiosis Linked to Colorectal Cancer Prevention
Source: Nutrients. 2020 Jun 7;12(6):1705. doi: 10.3390/nu12061705 (PMC7352482; doi:10.3390/nu12061705)
Supplement: Supplementary file 1 [file nutrients-12-01705-s001.pdf]

Table S1. Composition of experimental high fat diets

	coconut	***sunflower***	***EVOO***
**Diet component**		g/kg	
Custom diet mix[1]	666.6	666.6	666.6
Coconut oil	333.4	-	-
Sunflower oil	-	333.4	-
Extra virgin olive oil		-	333.4
		% kcal	
Protein	18	18	18
Carbohydrate	22	22	22
Fat	60	60	60
**Fatty acid composition**		g/100g total fatty acids	
Σ SFA[2]	90.4	7.7	16.04
Σ MUFA[3]	7.2	39.7	78.54
Σ PUFA[4]	1.9	52.7	4.46
**Key fatty acids**			
C8:0	5.8	-	-
C10:0	6.2	-	-
C12:0	46.2	-	-
C14:0	19.3	-	0.01
C16:0	10	5.1	13.61
C16:1	-	0.1	1.21
C18:0	2.9	2.6	2.42
C18:1	7.2	39.6	77.33
C18:2 ω6	1.9	52.6	3.64
C18:3ω3	-	0.1	0.82

[1]Custom diet mix supplied and formulated by ENVIGO®
[2]SFA: saturated fatty acids
[3]MUFA: monounsaturated fatty acids
[4]PUFA: polyunsaturated fatty acids

Table S2. Minority compounds found in EVOO

Minority compounds	
	mg/kg
Squalene	9701
Total tocopherols	378.2
α-Tocopherol	369.1
β-Tocopherol	3.6
γ-Tocopherol	5.6
δ-Tocopherol	<1.0
Tyrosol	2.5
	mg/kg tyrosol
Total Biophenols	265.1
Hydroxytyrosol	0.6
Oleocanthal	34
	mg caffeic acid/kg
Total Polyphenols	372

Table S3. PicoGreen-Based DNA Quantification and Quality control analysis

	PicoGreen-Based DNA Quantification assay	***Quality control***			
Sample	Concentration (ng/µl)	Secuencies number	Length	Total Mb	Mean quality
1	8,57	160.391	408,66	65,55	36,63
2	2,62	35.336	402,03	14,21	36,66
3	0,69	52.841	403,59	21,33	36,6
4	6,62	62.736	407,23	25,55	36,57
5	1,43	50.976	395,67	20,17	36,57
6	6,46	124.853	407,03	50,82	36,56
7	6,78	28.568	406,31	11,61	37,13
8	48,96	62.205	405,98	25,25	36,69
9	32,76	262.236	415,23	108,89	37,72
10	24	35.561	400,73	14,25	37,56
11	17,33	39.532	407,51	16,11	37,56
12	19,46	40.347	396,82	16,01	37,53
13	11,07	14.200	396,63	5,63	37,52
14	15,89	11.165	403,01	4,5	37,49
15	3,4	14.475	402,22	5,82	37,62
16	4,5	22.264	412,94	9,19	37,59
17	17,79	128.719	413,63	53,24	37,64
18	5,87	53.570	405,58	21,73	36,59
19	6,51	7.235	394,54	2,85	37,56
20	14,41	55.965	408,66	22,87	36,46
21	67,27	72.339	411,68	29,78	37,03
22	56,12	61.013	407,87	24,89	36,49
23	39,88	62.298	402,28	25,06	36,15
24	24,04	211.347	410,15	86,68	37,85
25	41,39	287.816	408,71	117,63	36,53
26	14,61	61.276	408,11	25,01	36,5
27	28,73	32.903	385,25	12,68	37,46
28	20,44	182.564	415,39	75,84	37,6
29	10,61	74.745	412,45	30,83	37,64
30	11,76	56.803	405,31	23,02	37,54
31	20,13	215.602	414,9	89,45	37,63
32	10,68	72.644	408,24	29,66	37,42

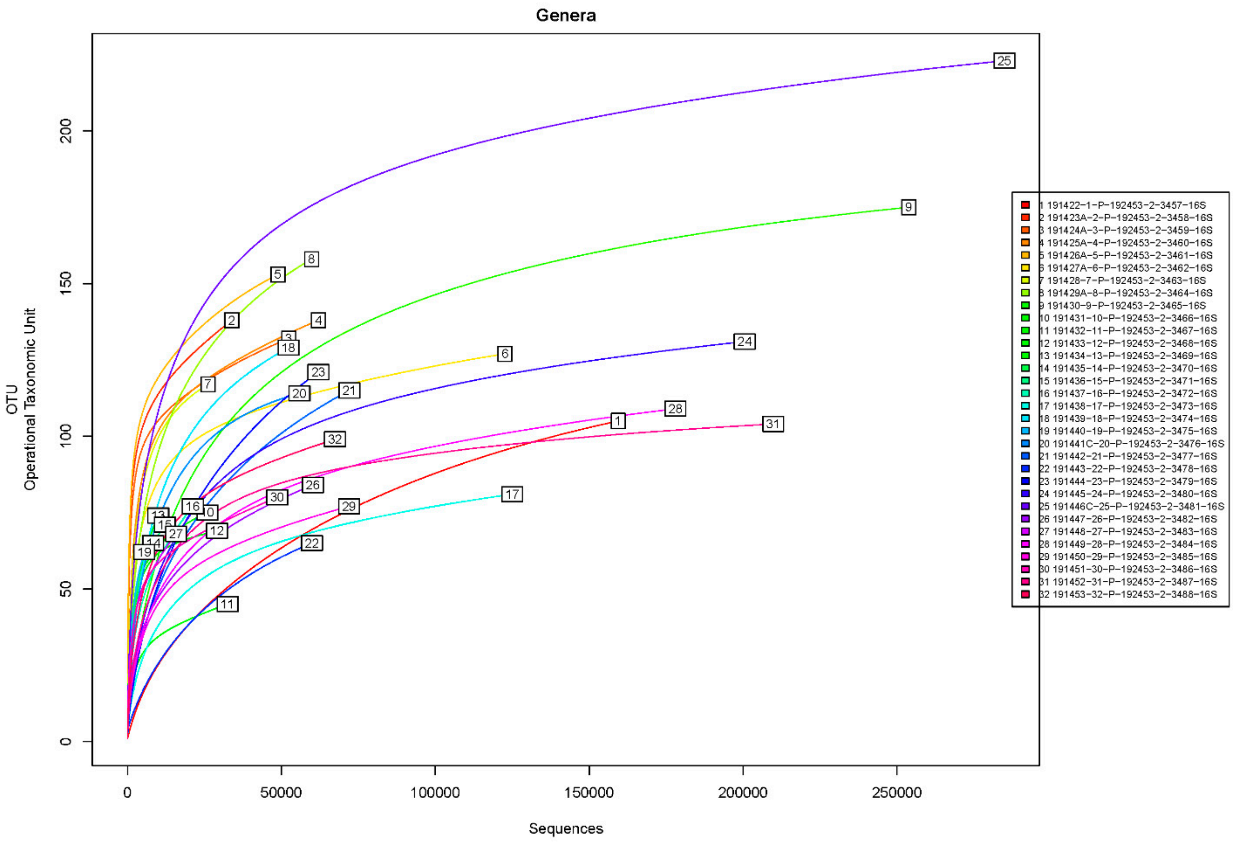

Figure S1. Rarefaction curves of the amplified samples for the detection of bacterial genera.
